# Assessing the neutralizing antibody and duration of RNA positivity from COVID-19 infected patients with immunocompromised diseases and pneumonia

**DOI:** 10.1186/s43556-024-00191-1

**Published:** 2024-07-28

**Authors:** Shuo Liu, Xuelian Wu, Ziteng Liang, Weijin Huang, Yufeng Xiong

**Affiliations:** 1 Division of HIV/AIDS and Sex-Transmitted Virus Vaccines, Institute for Biological Product Control, National Institutes for Food and Drug Control (NIFDC), Beijing, 100000 China; 2 Department of Laboratory Medicine, Nanfang Hospital, Southern Medical University, Guangzhou, 510515 China

Dear Editor,

Severe acute respiratory syndrome coronavirus 2 (SARS-CoV-2) has caused nearly 7 million deaths worldwide [1]. Nonhospitalized patients often develop asymptomatic or mild symptoms of COVID-19. However, the majority of hospitalized patients have an underlying disease, and some develop pneumonia [2]. According to reports, individuals in good health who have been infected with the omicron variant for a second time show a reduced immune imprint and produce broad-spectrum neutralizing antibodies (Nabs) against the omicron variant [3]. Therefore, the production of broad-spectrum Nabs against the omicron variant after a secondary infection in hospitalized patients warrants investigation. Some hospitalized patients are immunocompromised [4] and some patients develop pneumonia [5]. We compared the Nab titers and the time required to become negative for SARS-CoV-2 nucleic acid in these two groups of patients.

In this research, we assessed the cross-neutralization ability of serum samples taken from convalescent SARS-CoV-2 infected patients who were hospitalized at Southern Medical University South Hospital located in Guangdong Province, China. Using a pseudovirus assay (Supplementary Fig. S1), we examined the neutralization capacity of these samples against wild-type (WT) SARS-CoV-2 as well as various omicron subvariants. In total, 101 hospitalized patients were categorized into nine distinct groups according to their vaccination history, immune status, and pneumonia condition. They recruited participants who had been administered with either 1, 2, or 3 shots of inactivated WT vaccine prior to experiencing a subsequent breakthrough infection (BTI) (*n* = 17 in group 1, *n* = 25 in group 2, and *n* = 59 in group 3, respectively); Individuals who were immunocompetent and had received either 1, 2, or 3 doses of inactivated SARS-CoV-2 vaccine prior to being infected (*n* = 88 in group 4), as well as immunocompromised individuals who had received 1, 2, or 3 doses of the inactivated vaccine before undergoing a second BTI (*n* = 13 in group 5); Individuals diagnosed with lymphoid immune disease who had received 1, t2, or 3 doses of the inactivated vaccine prior to a second breakthrough infection (*n* = 8 in group 6); and individuals suffering from myeloid immune disease who had similarly received 1, 2, or 3doses of the inactivated vaccine before encountering a second breakthrough infection (*n* = 5 in group 7); Participants who did not have pneumonia and were administered either 1, 2, or 3 doses of inactivated SARS-CoV-2 vaccine prior to contracting the virus (*n* = 32 in group 8); and participants who had pneumonia and received either 1, 2, or 3 doses of inactivated SARS-CoV-2 vaccine before infection (*n* = 13 in group 9).

In all the groups, the NAb titers against omicron subvariant BA.5 were high, but were slightly lower (1.3–5.0-fold) against WT, XBB, XBB.1.5, and XBB.1.16 (Fig. S2a-c). Although the XBB strain caused second BTIs, immunoblotting showed that stronger Nab responses against the first BTI strain, BA.5, were induced.

Serum samples from group 3 (three vaccinations + second BTI) induced higher NAb titers against WT SARS-CoV-2 and all the omicron subvariants tested than did the samples from groups 1 and 2 (hospitalized patients vaccinated once or twice, respectively, + second BTI) (Fig. S2a-c). These results indicate that three doses of the vaccine induced higher NAb titers in hospitalized patients.

Serum samples from group 5 (vaccinated immunocompromised patients + second BTI) showed lower neutralization activity than samples from group 4 (hospitalized immunocompetent patients) (Fig. 1a). When the NAb titers against the XBB series of pseudoviruses were considered,

**Fig. 1:**
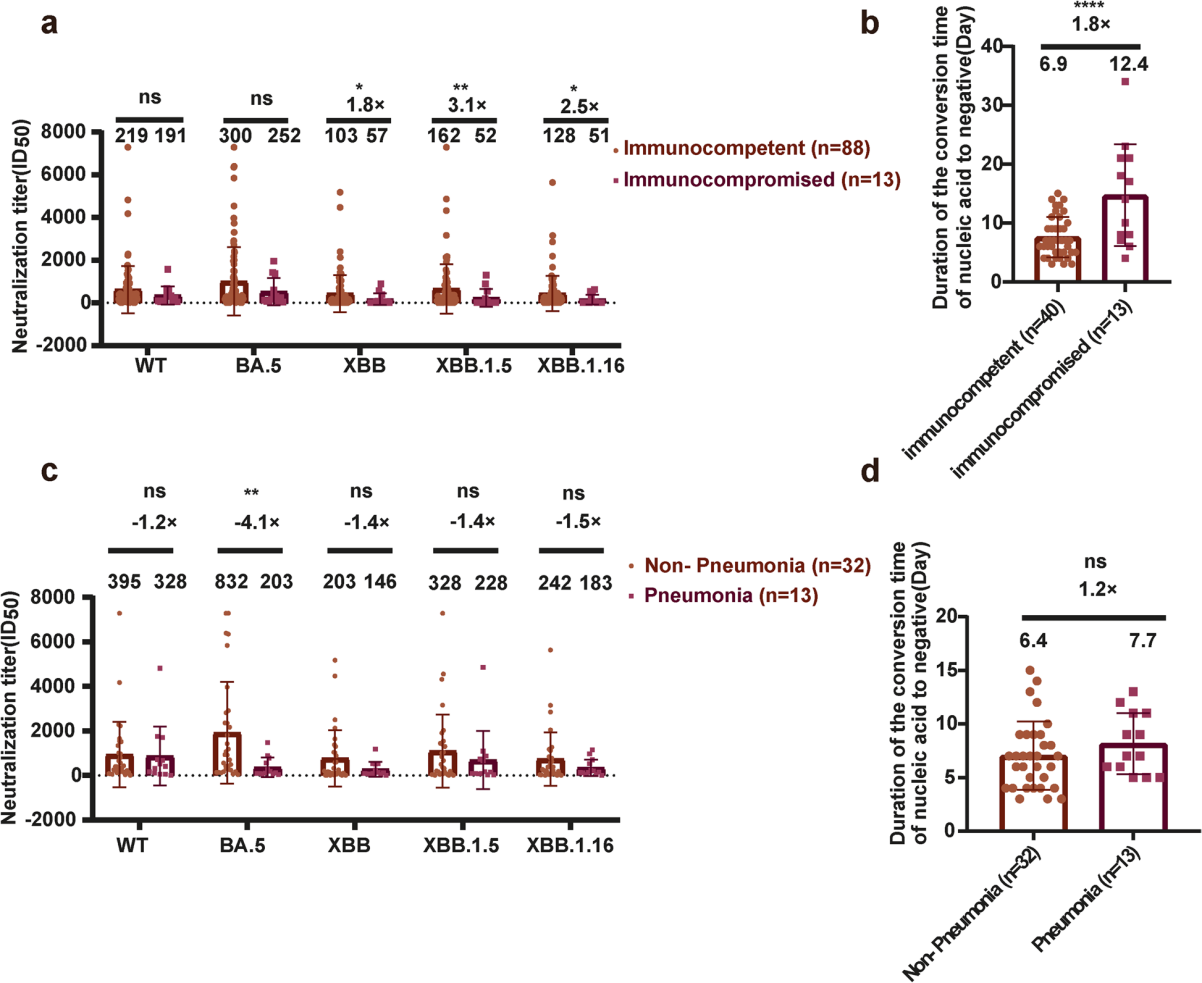
Neutralizing antibodies against SARS-CoV-2 variants in sera from convalescent hospitalized patients. **a**, **b** Comparison of neutralizing antibody titers and duration of positive nucleic acid between immunocompetent individuals and immunocompromised individuals. **c**, **d** Comparison of neutralizing antibody titers and duration of positive nucleic acid between non-pneumonia patients and pneumonia patients. To statistically analyze multiple sets of data, one or two-way ANOVA tests and Dunnett’s multiple comparisons test were utilized. The experimental data obtained from three repeated trials. The results are presented as means ± standard deviations (SD). Significance thresholds: *p < 0.05, **p < 0.01, ***p < 0.005, and ****p < 0.001

those of immunocompromised patients were approximately was 1/2–1/3 of the immunocompetent patients. The humoral immune response after BTI is influenced by the immune status of the patient, and the results of this study show that the NAb levels in immunocompromised individuals are lower than those in immunocompetent individuals.

We measured the periods over which some hospitalized patients were positive for SARS-CoV-2 nucleic acid. The average periods of nucleic acid positivity in the populations that received one, two, or three doses of vaccine were 7.14, 6.83, and 6.83 days, respectively (Fig. S2d). Although the period of positivity was shortest after three doses of vaccine, the differences were not statistically significant. However, the duration of nucleic acid positivity was related to the immune status of the patient. The average period of nucleic acid positivity was longer in immunocompromised individuals than in individuals with normal immune function (12.4 days and 6.9 days, respectively) (Fig. 1b). This immunocompromised population included individuals with lymphatic system disease (follicular lymphoma, acute lymphoblastic leukemia,) or myeloid system disease (myelodysplastic syndrome, diffuse large B-cell lymphoma, acute myeloid leukemia). When we recalculated the average period of nucleic acid positivity in patients with lymphatic system or myeloid system disease, it was up to 7.7 days in patients with myeloid system disease (group 8), but 16.7 days in those with lymphatic system disease (group 7) (Fig. S3c). Thus, the NAb titers of the lymphatic-immunodeficient individuals was 1/12–1/6 of the immunocompetent individuals within the three pseudoviruses(XBB, XBB.1.5, and XBB.1.16). However, there was no significant difference between the NAb titers of myeloid-immunodeficient individuals and immunocompetent individuals (Fig. S3a). It is possible that both T lymphocytes and B lymphocytes are damaged in patients with lymphatic system disease, thus affecting the clearance of the virus and the production of NAbs. In contrast, the maturation of B cells, but not that of T lymphocytes, is affected in patients with myeloid system disease, so the effects on the period of nucleic acid positivity and the level of the humoral immune response are not significant. After analyzing the medication usage in immunocompromised patients, we found that patients who underwent bone marrow transplantation and were treated with a combination of dexamethasone and anti-rejection immunosuppressants had higher levels of neutralizing antibodies. In contrast, patients who only received dexamethasone, without undergoing bone marrow transplantation, had lower levels of neutralizing antibodies (Fig. S3b). There was no significant difference in the duration of nucleic acid turning negative between the two groups of patients (Fig. S3d).

Among patients hospitalized with COVID-19, some had pneumonia. We separated the hospitalized patients into two groups: those without pneumonia (group 8) and those with pneumonia (group 9), and compared their levels of NAbs and periods of nucleic acid positivity. The average level of NAbs against BA.5 in the pneumonia patients was 1/4 of in the population without pneumonia (geometric mean titers: 203 and 832, respectively, *P* < 0.01) (Fig. 1c). This difference may be related to the protective effect of high titers of NAbs against pneumonia. Although the period of nucleic acid positivity was longer in pneumonia patients than in nonpneumonia patients, the difference was not statistically significant (Fig. 1d).

In summary, we detected an immune imprinting phenomenon in a SARS-CoV-2-infected population vaccinated with an inactivated vaccine who had experienced a second BTI. The population vaccinated with three doses of inactivated vaccine showed strongest antibody cross-neutralization activity after a second BTI. However, the titers of NAbs were lower in immunocompromised hospitalized patients than in immunocompetent patients. Notably, the NAb titer was significantly lower in patients with lymphatic immune deficiency than in immunocompetent patients or those with myeloid system disease. A higher NAb titer may contribute to the prevention of pneumonia. Taken together, these findings offer essential support to the basic data on the prevention and treatment of COVID-19.

### Supplementary Information

Supplementary Material 1.

## Data Availability

The data supported the results in this study are available from the corresponding author upon reasonable request.
