# Supplementary material for: Assessing the neutralizing antibody and duration of RNA positivity from COVID-19 infected patients with immunocompromised diseases and pneumonia
Source: Mol Biomed. 2024 Jul 28;5:29. doi: 10.1186/s43556-024-00191-1 (PMC11283440; doi:10.1186/s43556-024-00191-1)
Supplement: Supplementary file 1 — Supplementary Material 1. [file 43556_2024_191_MOESM1_ESM.docx]

**Assessing the neutralizing antibody and the duration time of nucleic acid positive of SARS-CoV-2 among the hospitalized COVID-19 infected patients with immunocompromised diseases and pneumonia**

Shuo Liu ^a^ , Xuelian Wu ^a^ , Ziteng Liang ^a^ , Weijin Huang ^a*^ , Yufeng Xiong ^b*^

*^a^Division of HIV/AIDS and Sex-transmitted Virus Vaccines, Institute for Biological Product Control, National Institutes for Food and Drug Control (NIFDC), Beijing;*

*^b^Department of Laboratory Medicine, Nanfang Hospital, Southern Medical University, Guangzhou 510515, China*

^*^Correspondence: Yufeng Xiong (xyuf9002@smu.edu.cn), Weijin Huang (huangweijin@nifdc.org.cn)

**Matericals**

Clinical Serum Samples: Serum samples were collected from Southern Medical University Nanfang Hospital, including 101 positive samples obtained from participants who were vaccinated with the anti-SARS-CoV-2 vaccine. All serum samples were pre-measured to obtain anti-SARS-CoV-2 NAbs titers using the pVNT method.

The background information of these samples

In the classification of vaccine immunity, there were 17 individuals who received a single-dose vaccine with a median age of 61.5 years, 47% male and 53% female. The median time interval from vaccination to infection was 427 days. For the two-dose vaccine group, there were 25 individuals with a median age of 54.4 years, 48% male and 52% female. The median interval between the first and second doses was 92 days, and the median interval from the second dose to infection was 570 days. In the three-dose vaccine group, there were 59 individuals with a median age of 52.1 years, 56% male and 44% female. The median interval between the first and second doses was 33 days, between the second and third doses was 193 days, and from the third dose to infection was 484 days.

There were a total of 13 immunocompromised patients, with 8 individuals having lymphatic immune diseases and 5 individuals having myeloid immune diseases. The gender distribution was 46% male and 54% female. Among the patients, there was 1 case of Follicular lymphoma, 2 cases of Acute lymphoblastic leukemia, 4 cases of Diffuse large B-cell lymphoma, 1 case of Vascular immune maternal lymphoma T-cell lymphoma, 3 cases of Acute myeloid leukemia, 1 case of Chronic myelogenous leukemia, and 1 case of Medulloblastoma.

There are 32 non-pneumonia patients, with a median age of 54.6 years, 56% male and 14% female; and 13 pneumonia patients, with a median age of 58.2 years, 62% male and 38% female.

This study was approved by the Southern Medical University Nanfang Hospital (Guangzhou, China) (ethics approval number: ChiCTR2300078127). All human serum experiments were performed according to The Code of Ethics of the World Medical Association (Declaration of Helsinki).

**Methods**

Pseudotyped SARS-CoV-2 variants

The Omicron variant gene of SARS-CoV-2 spike protein (GISAID: EPI_ISL_6590782.2) was optimized and synthesized using mammalian codons, and then cloned into pcDNA3.1 vector as described before^1^. Plasmids expressing S protein of WT, BA.5, XBB, XBB.1.5, XBB.1.16 SARS-CoV-2 variants were previously constructed^2^. The pseudotype SARS-CoV-2 variant based on VSV was transfected into 293 T cells (CRL-3216) with S protein expression plasmid and infected with G* Δ G-VSV (Kerafast, Boston). The titre of pseudovirus was evaluated by using Huh 7 (JCRB0403) cells through three times continuous dilution. Chemiluminescence signals were detected after cells and viruses were incubated at 37°C and 5% CO2 for 24 h. The Britellite Plus reporter gene assay system (PerkinElmer, Thermo Fisher Scientific) and the PerkinElmer Ensight photometer are utilized for signal acquisition. The detailed procedure has been described in our previous publication^1^.

Pseudotyped virus neutralization assay

Briefly, add 100 μl of samples 96-well plate and the samples were 1:30 diluted, followed by a 3-fold serial dilution. The diluted samples were mixed with 50 μl pseudotyped SARS-CoV-2 variants (1.3 × 10^4^ TCID_50_) in 96-well plates, respectively. The mixture was followed by incubation at 37°C for 1 h, and then mixed with Huh 7 cells (2–3 × 10^4^ cells/well) followed by incubation at 37°C in a humidified atmosphere with 5% CO2. The chemiluminescence signals in terms of relative luminescence unit (RLU) value was determined as described previously. The 50% inhibition dilution (ID_50_) was calculated using the Reed–Muench method^1^.

Statistical analysis

Data were analyzed with the GraphPad Prism 8.0 software (GraphPad, San Diego, CA). An unpaired two-tailed Student’s t-test was employed to compare two sets of data. To statistically analyze multiple sets of data, one or two-way ANOVA tests and Dunnett’s multiple comparisons test were utilized. The experimental data obtained from three repeated trials. The results are presented as means ± standard deviations (SD). Significance thresholds: *p < 0.05, **p < 0.01, ***p < 0.005, and ****p < 0.001.


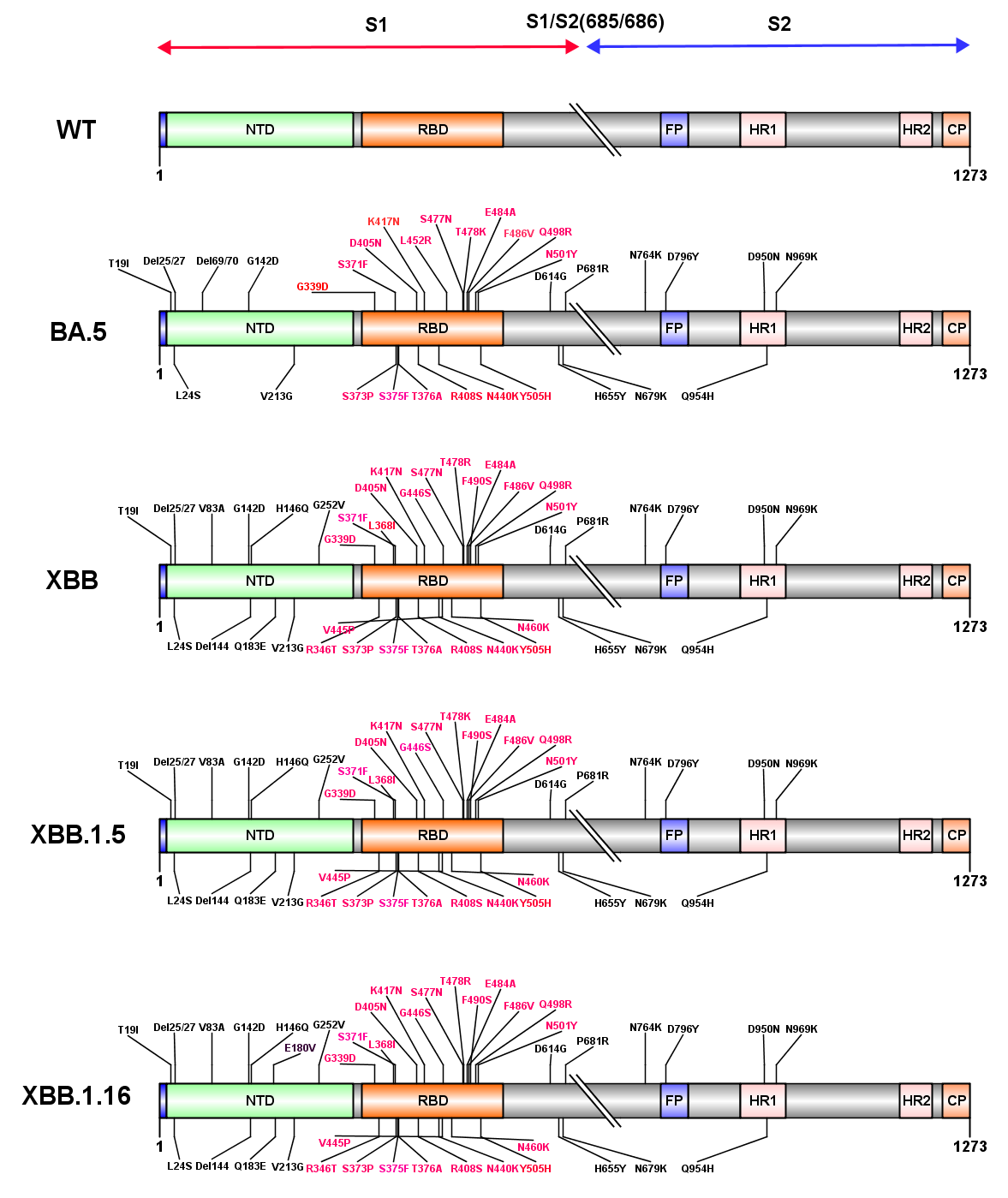


**Fig. S1** shows spike protein sequences for the BA.5, XBB, XBB.1.5 and XBB.1.5 variants; mutations as compared with the WT spike protein are shown, The red colour show the mutations of RBD.


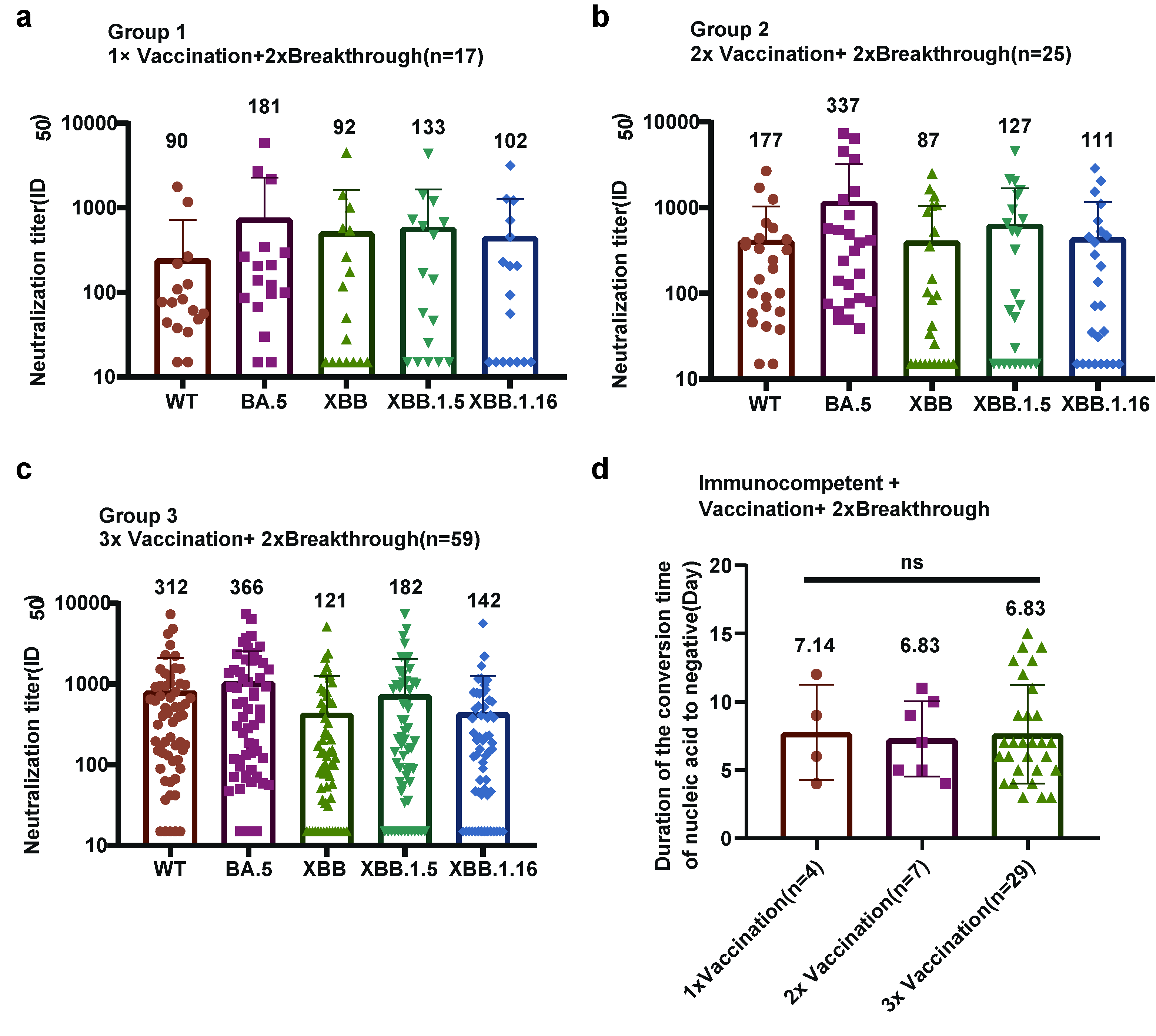


**Fig .S2** Neutralizing antibody titers against the WT strain and the Omicron variant, as well as the duration of positive nucleic acid in individuals after two breakthrough infections following different numbers of vaccine doses. a. Neutralizing antibody titers against the WT strain and the Omicron variant in individuals after two breakthrough infections following one dose of vaccine immunity. b. Neutralizing antibody titers against the WT strain and the Omicron variant in individuals after two breakthrough infections following two dose of vaccine immunity. c. Neutralizing antibody titers against the WT strain and the Omicron variant in individuals after two breakthrough infections following three dose of vaccine immunity. d. Duration of positive nucleic acid in individuals after two breakthrough infections with the WT strain and the Omicron variant following different numbers of vaccine doses. To statistically analyze multiple sets of data, one or two-way ANOVA tests and Dunnett’s multiple comparisons test were utilized. The experimental data obtained from three repeated trials. The results are presented as means ± standard deviations (SD). Significance thresholds: *p < 0.05, **p < 0.01, ***p < 0.005, and ****p < 0.001.


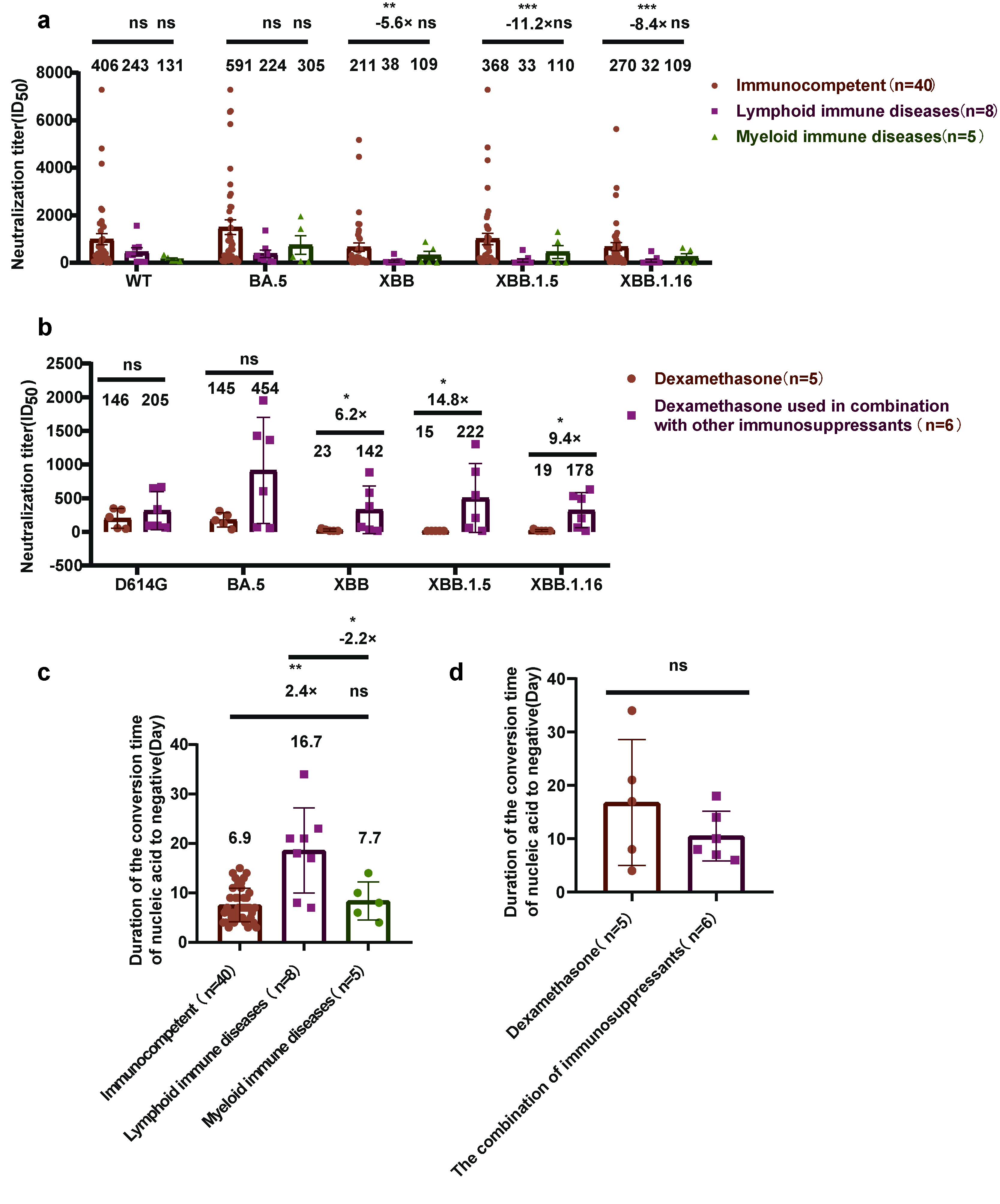


**Fig .S3** Comparison of neutralizing antibody titers and duration of positive nucleic acid in immunocompromised patients. a,c Comparison of neutralizing antibody titers and duration of positive nucleic acid in immunocompromised patients with myeloid and lymphoid immune deficiencies. b,d Comparison of neutralizing antibody titers and duration of positive nucleic acid in immunocompromised patients based on the types of immunosuppressants used. To statistically analyze multiple sets of data, one or two-way ANOVA tests and Dunnett’s multiple comparisons test were utilized. The experimental data obtained from three repeated trials. The results are presented as means ± standard deviations (SD). Significance thresholds: *p < 0.05, **p < 0.01, ***p < 0.005, and ****p < 0.001.

**Supplementary References**

1. Nie J, Li Q, Wu J, et al. Quantification of SARS-CoV-2 neutralizing antibody by a pseudotyped virus-based assay. *Nat Protoc.* 2020;15(11):3699-3715.

2. Li Q, Wu J, Nie J, et al. The Impact of Mutations in SARS-CoV-2 Spike on Viral Infectivity and Antigenicity. *Cell.* 2020;182(5):1284-1294 e1289.
